# Effect of 48-week pegylated interferon α-2a or nucleos(t)ide analogue therapy on renal function in Chinese patients with chronic hepatitis B

**DOI:** 10.1186/s12985-017-0712-x

**Published:** 2017-03-09

**Authors:** Ye Zhang, Wei-Lu Zhang, Xiao-Wen Pang, Lin-Xu Wang, Xin Wei, Chang-Xing Huang, Xue-Fan Bai, Shuai Han, Lin-Na Liu, Jian-Qi Lian

**Affiliations:** 1 Center for Infectious Diseases, Tangdu Hospital, Fourth Military Medical University, 569 Xinsi Rd, Xi’an, China; 2 Department of Epidemiology, School of Public Health, Fourth Military Medical University, Xi’an, China; 3 Department of Oncology, Tangdu Hospital, Fourth Military Medical University, Xi’an, China; 4 Department of Medical Quality Management, Tangdu Hospital, Fourth Military Medical University, Xi’an, China; 5 The First Brigade of Student, Fourth Military Medical University, Xi’an, China; 6 Department of Pharmaceutics, Tangdu Hospital, Fourth Military Medical University, 569 Xinsi Rd, Xi’an, China

**Keywords:** Chronic hepatitis B, Chronic kidney diseases, Antiviral, Drug, Mixed Linear Model

## Abstract

**Background:**

Controversy remains as to whether antiviral agents contribute to renal dysfunction in patients with chronic hepatitis B virus (HBV) infection. Thus, the aim of study was to analyze the changes in renal function of chronic hepatitis B (CHB) patients in response to anti-HBV therapy and the association with treatments.

**Method:**

We performed a retrospective observational cohort study to investigate factors associated with renal function in 249 Chinese CHB patients who were treated with pegylated interferon α-2a (PEG-IFN-α-2a) or nucleos(t)ide analogues for 48 weeks. Changes of estimated glomerular filtration rate (eGFR), which was computed with both the Chronic Kidney Disease Epidemiology Collaboration and the Modification of Diet in Renal Disease formulas, were tested by repeated measures One-way analysis of variance within groups. A linear mixed effects model for repeated measures was also used to evaluate the association between baseline information and eGFR changes over time in all enrolled patients. The model considered the baseline age, sex, HBV DNA, aminotransferase, blood urea nitrogen, treatment group, time, and group-by-time interaction as fixed effects and incorporated random effects for individual subjects.

**Results:**

The eGFR increased in patients given PEG-IFN-α-2a, decreased in patients given adefovir, but remained stable in patients given entecavir. Age and blood urea nitrogen were significant negative predictive factors for eGFR changes.

**Conclusion:**

In real-life study, PEG-IFN-α-2a therapy in CHB patients increased eGFR, thus may associate with renoprotective effects when compared with adefovir or entecavir therapies.

## Background

Chronic hepatitis B (CHB) is one of causes of chronic renal disease, mainly through deposition of immune complexes in the kidney [1]. In the area with high hepatitis B virus (HBV) prevalence, such as many within Asia-Pacific region, HBV-related membranous nephropathy and mesangiocapillary glomerulonephritis are closely correlated with end-stage renal diseases and renal replacement therapy [2, 3]. However, the mechanism of these renal dysfunctions has not been fully elucidated. The potential confounding factors include elder age, hypertension, diabetes mellitus, human immunodeficiency virus (HIV) co-infection, end-stage liver diseases, and nephrotoxic drugs [4].

HBV induced kidney diseases usually improved with inhibition of viral replication by anti-HBV agents [5]. Well accepted guidelines [6–8] for the management of HBV infection have been established in recent years. Therapeutic approaches for CHB consist of administration of interferon-α (IFN-α) or nucleos(t)ide analogues (NUCs). Five NUCs are currently available, including two nucleotide (adefovir [ADV] and tenofovir [TDF]) and three nucleoside (lamivudine [LAM], telbivudine [LdT], and entecavir [ETV]). Renal excretion with unchanged drugs is the primary route of elimination of NUCs [9]. Thus, all NUCs exist dose-dependent kidney toxicities by various mechanisms [10]. ADV treatment has been previously revealed to be associated with the impairment of renal function [11–13]. Decrease in estimated glomerular filtration rate (eGFR) was also found in TDF and ETV-treated patients [14]. In contrast, long-term LdT therapy was closely related to sustained improvement of renal function, particularly among patients with high risk of renal dysfunction, such as decompensated cirrhosis [15] and combination therapy with ADV [16]. However, controversy remains as to safety profile with findings either an increase or a decrease in eGFR during long-term and various classes of antiviral agents in real-life study. Furthermore, few studies focus on the safe renal profile of IFN-α, especially pegylated interferon α-2a (PEG-IFN-α-2a) which was recommended as first-line antiviral drugs by National Institute for Health and Clinical Excellence. Hence, the aim of this retrospective study was to assess the renal function and antiviral efficacy under PEG-IFN-α-2a and/or NUCs therapy in chronic hepatitis B. Known risk factors were also took into account to analyze the predictors for significant eGFR change.

## Methods

### Study design

We screened an integrated database which included 678 consecutive patients with chronic HBV infection who received PEG-IFN-α-2a (180 μg, subcutaneous injection weekly), ADV (10 mg, orally once daily), LdT (600 mg, orally once daily), ETV (0.5 mg, orally once daily) or combination of PEG-IFN-α-2a and ETV between December 2005 and March 2013 at a single unit in Center for Infectious Diseases, Tangdu Hospital. The enrolled patients met the following criteria: Diagnoses of CHB according to the standard of the Chinese National Program for Prevention and Treatment of Viral Hepatitis; absence of other hepatitis virus or HIV co-infection; absence of concurrently afflicted by decompensated liver cirrhosis (including ascites, hepatic encephalopathy, varicealbleeding, spontaneous bacterial peritonitis), liver failure, or hepatocellular carcinoma; absence of hypertension, diabetes mellitus, immunocompromised diseases, autoimmune diseases, solid cancer or leukemia. All patients included in this cohort underwent a follow-up evaluation every 12 weeks for a total of 48 weeks. Virological and biochemical assessments were performed as routine examination at every visit. The study protocol was approved by the Ethics Committee of Tangdu Hospital on May 2015 (Approval No. TDLL-201505-013). The data were collected on July and August, 2015, and we had access to information that could identify individual enrolled subjects during and after data collection.

### Virological and biochemical assessment

Serum HBV DNA was quantified by real-time polymerase chain reaction kit (PG Co Ltd, Shenzhen, Guangdong, China) with detection limit threshold of 2 log10 copies/mL. HBsAg, HBeAg, and anti-HBe was quantified using the ARCHITECT HBsAg, HBeAg, and anti-HBe reagent kit (Abbott GmbH & Co. KG, Wiesbaden, Germany), respectively. Serum biochemical assessments (including alanine aminotransferase [ALT], aspartate aminotransferase [AST], bilirubin, albumin, blood urea nitrogen [BUN], and serum creatinine [Cr]) were measured using an automatic analyzer (Hitachi 7170A, Hitachi Ltd, Tokyo, Japan) in Department of Clinical Laboratory Medicine of Tangdu Hospital.

### Evaluation of renal function

The eGFR was estimated by the following formulas based on Cr. The Chronic Kidney Disease Epidemiology Collaboration (CKD-EPI) calculation for eGFR (mL/min/1.73 m^2^) = 141 × min(Cr/κ, 1)^α^ × max (Cr/κ, 1)^-1.209^ × 0.993^Age^ × 1.018 (if female). κ is 0.7 for female and 0.9 for male. α is −0.329 for male and −0.411 for male [17]. The Modification of Diet in Renal Disease (MDRD) calculation for eGFR (mL/min/1.73 m^2^) = 186 × Cr^-1.154^ × Age^-0.203^ × 0.742 (if female) [18].

### Statistical analysis

The Chi-squared-test, One-way analysis of variance (ANOVA), or Kruskal-Wallis test was used to assess the differences in demographic and clinical variables among groups. All continuous variables were tested by repeated measures ANOVA. To evaluate the association between several variables and eGFR changes over time, a linear mixed effects model for repeated measures was used by SAS 9.4 with MIXED procedure. The model considered the baseline age (in years), sex, HBV DNA, ALT, AST, BUN, treatment group, time and group-by-time interaction as fixed effects and incorporated random effects for individual subjects. All *P* values are 2-sided, and the type I error was set as 5%.

## Results

### Baseline characteristics of enrolled patients

The cohort comprised 249 consecutive CHB patients with 48-week therapy and evaluation. Baseline characteristics for patients were shown in Table 1. There were no differences among groups in terms of age and sex. However, the distribution of eGFR, BUN, Cr, ALT, AST, and HBV DNA levels were remarkably different among groups. There were a total of 66 enrolled CHB patients with PEG-IFN-α-2a therapy, including 39 of treatment naïve patients and 27 of ETV-experienced patients. ETV experienced patients received more than 3 years therapy with ETV, and then switched to PEG-IFN-α-2a therapy. These patients were demonstrated with undetectable viral replication and HBeAg negative in the serum, and only four of them suffered with abnormal aminotransferase levels. All other 222 CHB patients were positive for HBeAg and treatment-naïve for NUCs or IFNs. The mean baseline eGFR was highest in patients with PEG-IFN-α-2a therapy and was lowest in patients with ADV treatment based on CKD-EPI calculation. Based on CKD-EPI formula, only seven patients revealed an eGFR less than 90 mL/min/1.73 m^2^, and no patients showed a baseline eGFR less than 60 mL/min/1.73 m^2^. Based on MDRD formula, sixteen patients showed an eGFR less than 90 mL/min/1.73 m^2^, with one patients with baseline eGFR of 59.58 mL/min/1.73 m^2^.

**Table 1:** Characteristics of 249 chronic HBV-infected patients treated with pegylated interferon α-2a or nucleos(t)ide analogues

Characteristics	ADV	ETV	LdT	PEG-IFN-α-2a (treatment naïve)	PEG-IFN-α-2a (ETV experienced)	*P* value
Patients (*n*)	72	58	53	39	27	
Age (year)	28.69 ± 8.09	30.29 ± 8.55	28.62 ± 8.73	27.33 ± 6.47	30.11 ± 9.81	0.579^a^
Male sex [*n* (%)]	59(81.94%)	48(82.76%)	35(66.04%)	31(79.49%)	22(81.48%)	0.904^b^
HBV DNA (log10 copies/ml)	7.40 ± 0.94	7.58 ± 1.53	7.87 ± 1.32	8.17 ± 1.37	<2	0.019^a^
ALT (U/L)	172.5 ± 173.6	131.9 ± 128.6	176.1 ± 145.7	162.6 ± 92.12	31.32 ± 26.32	<0.0001^c^
AST (U/L)	95.44 ± 72.68	97.69 ± 152.4	122.7 ± 133.8	98.10 ± 55.33	29.25 ± 10.92	<0.0001^c^
BUN (mmol/L)	4.45 ± 0.98	4.37 ± 1.10	4.68 ± 1.27	4.87 ± 1.16	5.16 ± 1.34	0.025^a^
Cr (mg/dl)	0.93 ± 0.11	0.75 ± 0.12	0.74 ± 0.13	0.75 ± 0.11	0.77 ± 0.13	<0.0001^a^
CKD-EPI eGFR (mL/min/1.73 m^2^)	106.3 ± 13.99	121.2 ± 10.38	121.5 ± 11.21	123.1 ± 9.64	120.1 ± 11.50	<0.0001^a^
MDRD eGFR (mL/min/1.73 m^2^)	100.00 ± 14.68	127.7 ± 22.13	126.4 ± 20.96	127.4 ± 20.16	125.1 ± 22.69	<0.0001^a^

Values are presented as mean ± SD or *n* (percentage). ^a^One-way ANOVA test. ^b^Chi-squared test. ^c^ Kruskal-Wallis test

### Virological, biochemical, and serological responses

**Fig. 1:**
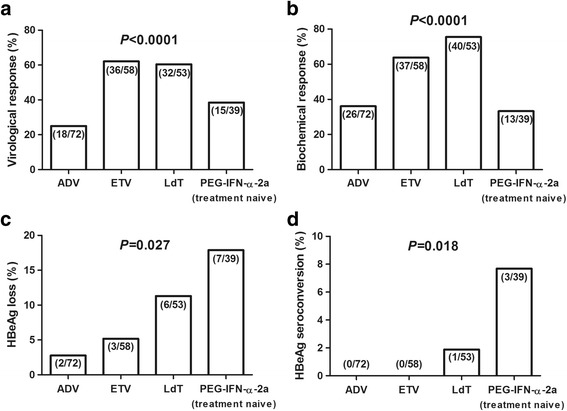
The rates corresponding to virological, biochemical, and serological responses to anti-HBV agents. **a** Rate of virological response (undetectable HBV DNA) at 48 weeks of therapy. **b** Rate of biochemical response (ALT normalization) at 48 weeks of therapy. **c** Rate of serologic response (HBeAg loss) at 48 weeks of therapy. **d** Rate of serologic response (HBeAg/anti-HBe seroconversion) at 48 weeks of therapy

The HBV DNA decreased in CHB patients received anti-HBV therapy. Patients who switched to PEG-IFN-α-2a therapy demonstrated continuous inhibition of viral replication during treatment. Furthermore, greater proportions of patients with ETV (62.1%) and LdT (60.4%) therapy showed significantly higher virological responses (VR) compared with naïve patients received ADV (25.0%) or PEG-IFN-α-2a (38.5%) at 48 weeks of therapy (*P* < 0.0001, Fig. 1a). At 48 weeks of therapy, a total of 21 patients (14 of ADV, 1 of ETV, and 6 of LdT therapy) revealed virological breakthrough, which was defined as an increase in HBV DNA levels to greater than 1 log10 copies/ml from nadir on at least two consecutive occasions. Direct sequencing demonstrated genotypic resistance to NUCs in 15 patients within those who suffered with virological breakthrough. Four ETV-experienced patients who revealed elevated ALT at baseline achieved biochemical response (BR) with normal ALT levels at 48 weeks of therapy. BR rates showed similar trends to VR among other four groups. Both ETV (63.8%) and LdT (75.5%) therapy revealed remarkable higher BR rates compared with patients received ADV (36.1%) or PEG-IFN-α-2a (33.3%) at 48 weeks of therapy (*P* < 0.0001, Fig. 1b). Moreover, greater proportions of patients who received PEG-IFN-α-2a (17.9%) and LdT (11.3%) therapy showed higher HBeAg loss rates compared with ADV (2.78%) or ETV (5.17%) therapy at week 48 (*P* = 0.027, Fig. 1c). Meanwhile, Four patients (1 in LdT and 3 in PEG-IFN-α-2a treatment) revealed HBeAg/anti-HBe seroconversion (Fig. 1d). However, HBsAg loss was not observed in NUCs-treated patients. One treatment-naïve and four ETV-experienced patients with PEG-IFN-α-2a therapy demonstrated HBsAg loss during therapy.

### Maintenance of eGFR improvement in PEG-IFN-α-2a therapy for CHB

**Fig. 2:**
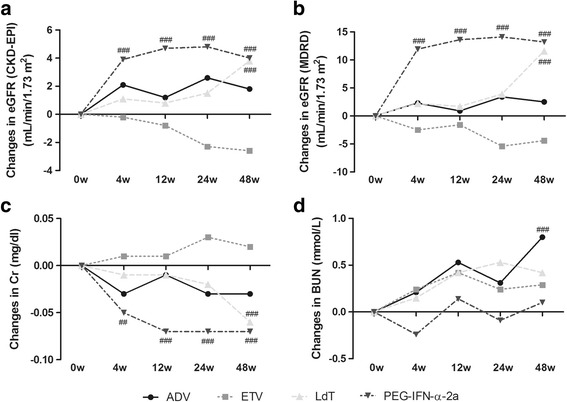
Evolution of renal function by anti-HBV agents therapy over 48 weeks. **a** Changes of eGFR as calculated by CKD-EPI formula. **b** Changes of eGFR as calculated by MDRD formula. **c** Changes of serum Cr. **d** Changes of BUN. “##” symbol indicated *P* < 0.01, and “###” symbol indicated *P* < 0.001

Based on the MDRD formula, a total of eighteen patients (14 receiving ADV therapy and 4 receiving ETV therapy) demonstrated renal dysfunction after anti-viral therapy. The changes in renal function (including BUN, Cr, and eGFR) were evaluated using repeated measures ANOVA which represented the matched values in different time points. Results with CKD-EPI and MDRD equations were comparable for eGFR changes during the 48 weeks of therapy. There were no significant differences in eGFR during either ADV or ETV treatment based on CKD-EPI (Fig. 2a) and MDRD formula (Fig. 2b). As expected, renal function steadily improved in LdT-treated CHB patients, and markers for renal function were improved at week 48 for patients with LdT therapy [eGFR (CKD-EPI) changes: +3.8 mL/min/1.73 m^2^, *P* = 0.0006, Fig. 2a; eGFR (MDRD) changes: +11.6 mL/min/1.73 m^2^, *P* = 0.0004, Fig. 2b]. Interestingly, eGFR increased rapidly at week 12 [eGFR (CKD-EPI) changes: +3.9 mL/min/1.73 m^2^, *P* < 0.0001, Fig. 2a; eGFR (MDRD) changes: +11.9 mL/min/1.73 m^2^, *P* < 0.0001, Fig. 2b], and remained in relatively high levels in all 66 patients (39 of treatment naïve and 27 of ETV-experienced) with PEG-IFN-α-2a therapy. eGFR achieved 125.9 ± 11.37 mL/min/1.73 m^2^ (CKD-EPI) and 140.0 ± 24.06 mL/min/1.73 m^2^ (MDRD) at the end of PEG-IFN-α-2a therapy, respectively. Serum Cr revealed similar improved trends with eGFR changes (Fig. 2c). BUN level increased with mean changes of +0.85 mmol/L in ADV-treated patients at week 48 (Fig. 2d).

**Fig. 3:**
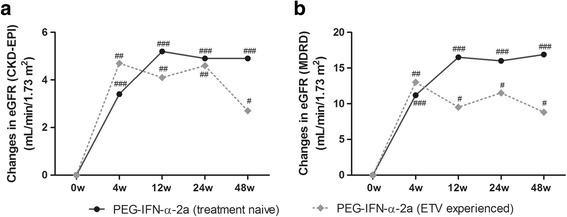
Evolution of eGFR by PEG-IFN-α-2a therapy in treatment-naïve patients or ETV-experienced CHB patients over 48 weeks. **a** Changes of eGFR as calculated by CKD-EPI formula. **b** Changes of eGFR as calculated by MDRD formula. “#” symbol indicated *P* < 0.05, “##” symbol indicated *P* < 0.01, and “###” symbol indicated *P* < 0.001

We further investigated the eGFR changes in treatment naïve and ETV-experienced patients with PEG-IFN-α-2a therapy. A steady increase in eGFR from baseline was observed in both subgroups. There was a remarkable elevation of eGFR at week 12 and continuously improved [treatment-naïve: eGFR (CKD-EPI) changes: +4.9 mL/min/1.73 m^2^, *P* < 0.0001, Fig. 3a; eGFR (MDRD) changes: +16.9 mL/min/1.73 m^2^, *P* < 0.0001, Fig. 3b. ETV-experienced: eGFR (CKD-EPI) changes: +2.7 mL/min/1.73 m^2^, *P* = 0.005, Fig. 3a; eGFR (MDRD) changes: +8.8 mL/min/1.73 m^2^, *P* = 0.0059, Fig. 3b]. Moreover, there were no differences in eGFR levels in each observation points in these two subgroups (*P* > 0.05).

### Predictors of significant eGFR change

We entered all variables, including treatment group time and group-by-time interaction as fixed effects and incorporated random effects in the linear mixed model accounting for repeated measures. Previous studies have been demonstrated that LdT therapy was associated with the improvement of renal function in CHB patients [15, 16, 19–22]. Furthermore, repeated measures ANOVA also showed an increased eGFR in LdT-treated patients in our study. Thus, LdT therapy was set as reference in this model. Results with CKD-EPI and MDRD equations were also comparable for the predictors of eGFR changes. We found that age, BUN, and ADV administration were significant predictors for decrease eGFR over time (Table 2). Among these variables, ADV administration was most capable of predicting eGFR decreases in CHB patients [estimated value of -14.522 (CKD-EPI) or −27.352 (MDRD), *P* < 0.0001]. Interestingly, treatment with PEG-IFN-α-2a in treatment-naïve patients was observed to be positively influenced eGFR values over time with statistically significant [estimated value of 2.766 (CKD-EPI) or 7.365 (MDRD), *P* = 0.035 and =0.036, respectively]. Furthermore, The changes of eGFR over time was not significantly associated with the reduction in both HBV DNA and aminotransferase.

**Table 2:** Predictors of eGFR changes

	eGFR (CKD-EPI)			eGFR (MDRD)		
	Estimate	Standard error	*P* value^a^	Estimate	Standard error	*P* value^a^
Age	−0.907	0.055	<0.0001	−0.933	0.122	<0.0001
Sex	1.410	1.105	0.203	4.916	2.473	0.048
HBV DNA	0.211	0.214	0.325	0.502	0.573	0.382
ALT	−0.006	0.004	0.102	−0.014	0.009	0.146
AST	0.006	0.005	0.254	0.014	0.013	0.296
BUN	−0.492	0.107	<0.0001	−1.028	0.266	0.0001
ADV	−14.522	1.397	<0.0001	−27.352	2.759	<0.0001
ETV	1.595	1.430	0.266	1.963	3.640	0.590
PEG-IFN-α-2a	2.766	1.306	0.035	7.365	3.496	0.036

^a^Results from the linear mixed effects model for repeated measures. LdT therapy was set as reference

## Discussion

HBV replication was directly associated with chronic pathological injury in kidney since HBV DNA could be detected in renal tubular cells of HBV related glomerulonephritis [23]. Thus, chronic hepatitis B increased the risk of end-stage renal diseases [24]. The current study was designed to assess renal function of CHB patients who were treated with PEG-IFN-α-2a or NUCs. A novel and important finding was that eGFR improved significantly in patients with PEG-IFN-α-2a therapy over 48 weeks. While LdT treatment was known to reveal the renal protective effect, ADV therapy showed a strong nephrotoxicity based on the eGFR decrease using the linear mixed effects model for repeated measure. The eGFR remained stable in patients with ETV treatment. Age and BUN were notable negative predictive factors for eGFR changes.

To the best of our knowledge, this is the first study on the effect of renal function with pegylated interferon therapy for HBV monoinfection. Remarkable elevations in eGFR were observed in both treatment-naïve and ETV-experienced CHB patients who received PEG-IFN-α-2a monotherapy. Interestingly, linear mixed effects model for repeated measures in the individual variations of eGFR also indicated the renoprotective function in PEG-IFN-α-2a-treated patients. Our results were in line with a previous study showing an increase in eGFR for patients with hepatitis B/D virus co-infection who received 48-week PEG-IFN-α-2a therapy [13]. The elevation of eGFR in LdT-treated patients was also consistent with several previous studies [15, 16, 20]. Moreover, the improvement of renal function in LdT based therapy was not remarkably associated with inhibition of HBV replication [15, 16], and our study in PEG-IFN-α-2a based treatment demonstrated a similar trend because the baseline HBV DNA levels in all of the ETV-experienced patients were under the limitation of detection from baseline to 48 weeks of therapy. Furthermore, it is generally elucidated that deposition of immune complexes of HBV antigens and host antibodies mediate most glomerular injuries [25]. More recent study on HBV-associated membranous nephropathy revealed that the percentage of CD4^+^CXCR5^+^ follicular T helper (Tfh) cells was negatively correlated with the value of eGFR [26]. Li et al. [27] also indicated that circulating CD4^+^CXCR5^+^ Tfh cells contributed to LdT-induced HBeAg seroconversion. This might indicate that the increase in eGFR was a direct beneficial effect from PEG-IFN-α-2a itself rather than an indirect effect by suppression of viral replication. It was reported that both PEG-IFN-α-2a and LdT demonstrated immnunomodulatory properties to control viral replication by activation of cellular and humoral immunity [28] and suppression of negative regulators [29]. Thus, we assumed that the immnunomodulatory role of PEG-IFN-α-2a and LdT may partially contribute to the increase of eGFR. However, the specific mechanisms by which PEG-IFN-α-2a and LdT exert their renoprotective effects were still unclear and remains to be clarified in future studies.

Mederacke et al. [13] revealed a decrease in eGFR during PEG-IFN-α-2a/ADV combination and ADV monotherapy for hepatitis B/D virus co-infection. We showed that ADV administration was most capable negative predictor for eGFR decrease for HBV monoinfection, consistent with the previous studies in different ethnic origins [4, 11–13, 20]. The nephrptoxicity of ADV was partly due to the inhibition of mitochondrial DNA replication during renal excretion [30], which leaded to dysfunction of mitochondrion and potentially caused clinical adverse events [9]. Meanwhile, TDF, which was also a nucleotide analogue as ADV, showed different safety renal profiles in several previous studies. An increase in serum Cr of more than 0.5 mg/dL in fewer than 1% of patients during 3-year TDF therapy [31], and renal impairment was detected in response to TDF treatment [4]. Significant elevation of serum Cr was commonly found in both ETV and TDF treatments in another study [14]. However, TDF was just approved for CHB treatment in China in June, 2014. There was no follow-up data for patients with TDF therapy for analysis in the present study. Previous study indicated similar risk of renal events in CHB patients with TDF or ETV treatment, showing that increase in serum Cr was more frequent with ETV than TDF [14]. Our results demonstrated minor kidney dysfunction for ETV monotherapy. Further studies on the changes in eGFR in patients with combination therapy of PEG-IFN-α-2a and NUCs should be performed to investigate the predominant renoprotective or impairment effects of anti-HBV agents.

This study has several limitations. This was a retrospective analysis of renal function by evaluation of eGFR, although data were derived from prospective study and all patients were well followed-up during the observational period. The enrolled patients was relatively young with most patients less than 50 years old, and only the baseline eGFR levels in ADV treated patients was low. Sixteen ADV treated patients were in CKD stage 2 or 3 with eGFR less than 90 mL/min/1.73 m^2^ at baseline based on MDRD formula. Furthermore, The limited number of enrolled patients and relatively short observational time for 48 weeks may also represent restrictions of our study. Thus, we used a linear mixed effects model for repeated measure to evaluate the individual variations of eGFR. Furthermore, no routine urine tests were performed during the study. Levels of serum Cr and changes of eGFR are late markers of renal impairment [4], which presumably secondarily after proximal tubular dysfunction. Nucleotide such as TDF and ADV tend to be more harmful to tubular than glomerular cells in both HBV and HIV infection [9, 32]. HBV infection could also induce subtle urinary abonormalities (e.g. proteinuria andhaematuroa) without obvious eGFR decrease in early stage. Thus, the exact impact of specific tubular toxicity of anti-HBV agents cannot be reliably appreciated.

## Conclusion

In conclusion, our results provided the evidence that PEG-IFN-α-2a therapy in CHB patients increased eGFR, thus may associated with renoprotective effects when compared with ADV or ETV therapies in real-life study. The mechanisms underlying the beneficial effects remain to be further investigated.

## Abbreviations

ADV: Adefovir
ALT: Alanine aminotransferase
AST: Aspartate aminotransferase
BUN: Albumin, blood urea nitrogen
CHB: Chronic hepatitis B
Cr: Creatinine
eGFR: Estimated glomerular filtration rate
ETV: Entecavir
HBV: Hepatitis B virus
HIV: Human immunodeficiency virus
IFN-α: Interferon-α
LAM: Lamivudine
LdT: Telbivudine
NUCs: Nucleos(t)ide analogues
PEG-IFN-α-2a: Pegylated interferon α-2a
TDF: Tenofovir
